# Effects of skin moisturization on various aspects of touch showing differences with age and skin site

**DOI:** 10.1038/s41598-023-44895-w

**Published:** 2023-10-20

**Authors:** Mariama Dione, Roger Holmes Watkins, Jean-Marc Aimonetti, Roland Jourdain, Rochelle Ackerley

**Affiliations:** 1 Aix Marseille Univ, LNC (Laboratoire de Neurosciences Cognitives – UMR 7291), CNRS, Marseille, France; 2 L’Oréal Research & Innovation, Aulnay-sous-Bois, France

**Keywords:** Neuroscience, Psychology

## Abstract

The human body is encompassed by a thin layer of tissue, the skin, which is heterogenous and highly specialized to protect the body and encode interactions with the external world. There is a fundamental scientific drive to understand its function, coupled with the need to preserve skin as we age, which impacts on our physiological and psychological well-being. In the present study, we aimed to define differences in touch perception between age groups and with skin cream application. We investigated touch on the finger, the forearm and cheek in younger (20–28 years, n = 22) and older (65–75 years, n = 22) females. We measured skin hydration, touch detection, finger spatial discrimination, forearm tactile pleasantness together with electrodermal activity, and perceptual ratings about cream use, skin dryness, and cosmetic habits. Glabrous finger skin became drier and touch performance was impaired with age, but these aspects were preserved in hairy skin. Skin moisturization immediately increased hydration levels, but did not significantly change touch perception. We also found that touch appreciation increased with age. We conclude that reduced finger capacity may impact self-evaluation of the skin and that long-term skin care strategies should focus on hydrating the hand to preserve touch capacities.

## Introduction

The skin is a complex, heterogenous sensory organ that forms a thin barrier around the body, serving a protective role against external aggressors and the sensing of interactions we have with our environment and others. Touch makes us aware of moment-to-moment contact on our body, which is accompanied by affective and emotional evaluations. Sensory innervation differs all over the body, depending on the skin type (glabrous, hairy), the receptors present, and their density. Further, skin changes over time produce effects that can be seen and felt. Aging has profound consequences for the skin, including visible anatomical changes, reduction in regulatory functions (e.g. sweat secretion), and somatosensory decline^1^. However, there are large inter-individual differences and some individuals exhibit less touch deterioration^2^. In our modern lives, there is increased awareness and concern for skin and its preservation, to counteract these issues^3^. This drives innovation in skin care and cosmetics, which can increase physiological and psychological well-being^4^.

The experience of touch gives rise to sensations concerning both discriminative/sensory aspects (e.g. vibration, hardness) and affective/emotional aspects (e.g. pleasant, arousing^5^). Tactile acuity and discrimination capacities are underpinned by fast-conducting, myelinated Aβ mechanoreceptive afferents that send precise information to the brain about the timing, force, direction, and duration of touch, as well as additional information, such as texture and compliance^6,7^. Much research has focused on the glabrous skin of the hands, which we primarily use to explore our world, and the types and density of mechanoreceptors present in glabrous skin offer high spatiotemporal tactile sensitivity, acuity, and discrimination, all of which are required for dexterous manipulation^6,8–10^. The hairy skin of the face is also exceptionally sensitive to touch, showing very high tactile sensitivity and discrimination, which is often better than on the hands^11–13^. This highlights the importance of touch signals from the face, which like the hands, is more openly exposed to environmental stressors (e.g. weather conditions). Thus, it is of interest to compare these regions directly, considering their importance (e.g. large cortical areas are devoted to the hands and face, their respective importance in object manipulation and communication), yet different location, use, structure, and innervation.

Affective touch is also very important in our lives, providing us with emotional meaning to touch. Affective touch awareness is thought to be conveyed by Aβ mechanoreceptors, but the positive aspects appear to be influenced by slowly-conducting, unmyelinated C-tactile (CT) afferents^14,15^. CT afferent signals arrive in the brain with a relative delay, which likely modulates and reinforces gentle touch^16^. CT afferents are present in hairy skin, have high densities on the arm^17^, and are readily found on the face^18^. Recent evidence indicates CTs are also present in glabrous hand skin, albeit sparsely^19^. Tactile spatial discrimination is generally worse in hairy skin, due to lesser innervation of mechanoreceptors, but is nevertheless highly sensitive to touch^20,21^ and is more implicated in receiving touch, such as affective touch from others^22^. Affective touch has a multitude of beneficial health implications and plays an important part in social interactions and communication^22^. It can aid in the relief of stress and depression and is associated with changes in autonomic functions, such as a lower heart rate and a decrease in blood pressure^23^. Thus, it is pertinent to investigate how both discriminative and affective aspects of touch come together and can be manipulated to increase tactile enjoyment, such as in using a cosmetic product.

Our sense of touch changes with age and there exists a dichotomy between discriminative tactile function and the perceived pleasantness of touch with aging (cf.^21,24^). Tactile sensitivity and acuity generally decrease with age, with changes in sensory nerve innervation and anatomical changes in the skin^8^. There is approximately a 5% decrease in mechanoreceptive afferents per decade in adults^25^, where by early middle age, there is already substantial afferent loss, although the lower limbs tend to be most affected^21^. However, tactile decline in aging is variable and tactile discrimination is preserved in some older people, even when there are biomechanical reductions in skin capacity^2^. The skin becomes drier, affecting its elasticity and reducing the ability to sense the friction exerted on touched surfaces, as well as to discriminate between surfaces^2,26^. It is not clear whether the effects of skin dehydration are similar at hairy skin level compared to the glabrous skin. To combat the deterioration of anatomical skin changes with aging, tactile acuity can be partially restored after hydration of the skin with a moisturizer^2,27,28^. Contrary to discriminative touch, affective touch does not seem to be impaired and may become even more hedonic as we age, although the mechanism behind this is unknown^24^.

In the present study, we aimed to draw together these themes to investigate touch differences between glabrous and hairy skin, changes with aging, and whether the application of a cosmetic cream modifies these factors. To this end, we used different touch tests, including self-reports, skin water content, tactile detection sensitivity, finger spatial tactile discrimination, forearm tactile pleasantness, and sensory ratings, to explore a wide range of touch facets in a younger group (20–28 years) and older group (65–75 years) of female participants. We hypothesized that there would be inherent differences between the skin sites investigated (finger, forearm, cheek), based on the anatomical structure and physiological innervation of the skin and that there would be increased variability with age^2^. We also applied a widely available, marketed cosmetic cream to the skin to investigate whether this changed these measures, predicting an improvement on tactile tests.

## Results

All data are available at: https://osf.io/znm2r/.

### Self-reported measures: skin classification and moisturizer use

**Figure 1:**
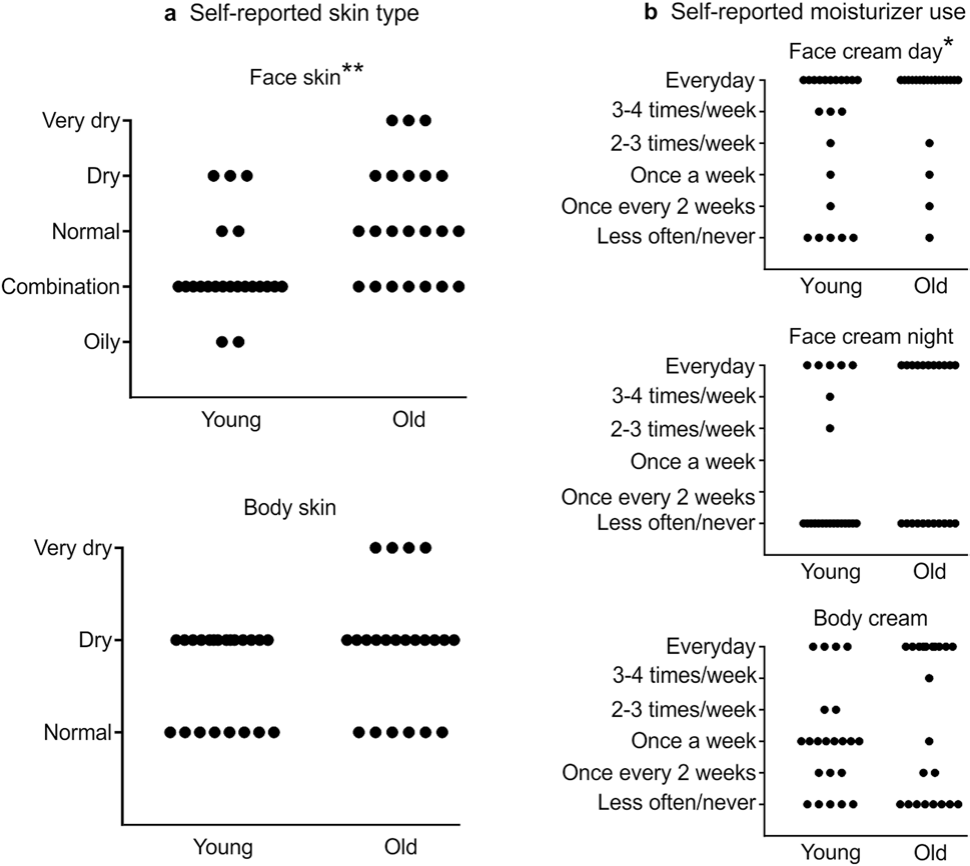
Self-perceived skin types and habitual moisturizer use for the younger and older groups. For the younger (n = 22) and older (n = 22) groups, their (**a**) self-reported face skin type is shown at the top left and body skin type on the bottom left. (**b**) Frequency of self-reported moisturizer use for facial day creams (top right), facial night creams (middle right), and body creams (bottom right) for the same participants. The frequencies of each measure are shown. Mann–Whitney tests where significant, are denoted on the title of each sub-figure as *p < 0.05, **p < 0.01.

We asked participants about their skin classification (oily–very dry) and routine cosmetic use, to gain information about the perception of their face and body skin, and their habits related to skin care. There was a significant difference between the self-reported face skin type between age groups (Mann–Whitney U = 123, p = 0.003). The median value for young participants was ‘combination’ (i.e. oily nose and forehead; n = 22) and in older participants was ‘normal’ (n = 22), thus overall, the older group found that they had drier facial skin (Fig. 1A, top). The median value for the self-reported body skin type in both young and old participants was ‘dry’ and there was no significant difference between these (Mann–Whitney U = 192, p = 0.212; Fig. 1A, bottom). Regarding self-reported moisturizer use, most participants used a face day cream every day, but this was significantly higher in the older group (Mann–Whitney U = 165, p = 0.034; Fig. 1B, top). For night cream use on the face, the results were split in both groups, where typically, participants either used a night cream every day or almost never, thus, in part due to this split, there was no significant difference between the age groups (Mann–Whitney U = 187, p = 0.156; Fig. 1B, middle). Concerning body cream use, the results were widespread across both age groups, showing no significant differences (Mann–Whitney U = 221, p = 0.623; Fig. 1B, bottom).

### Physical skin characteristics: water content

**Figure 2:**
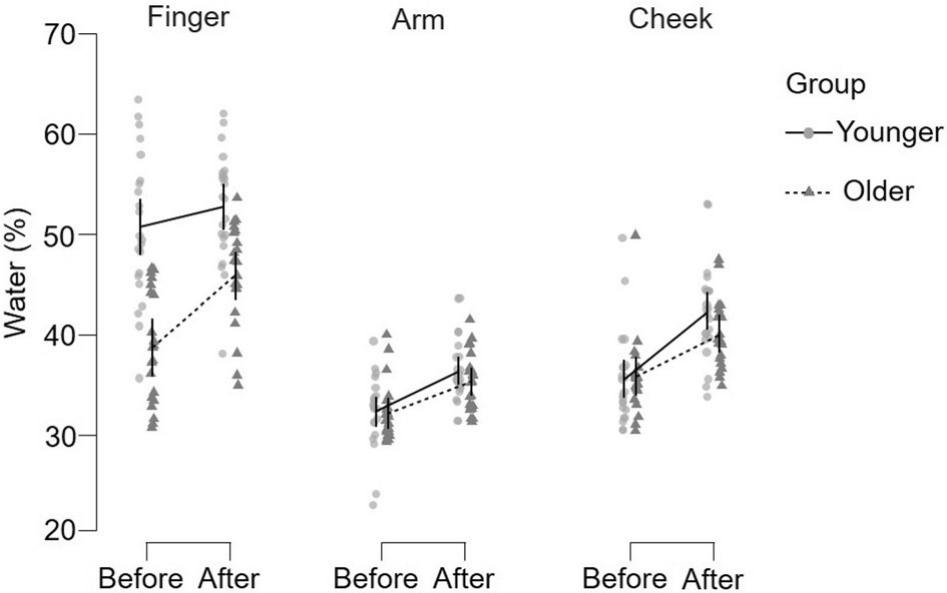
Skin water content between skin sites, over age groups, and with cream application. The percentage of skin water content is shown for each of the three skin sites tested (from left to right: finger, arm, cheek), where for each site, the water content before and after cream application is shown, for individuals in the younger group (n = 22, filled lighter gray circles, with a continuous line showing the means before-after cream application) and older group (n = 21, filled darker gray triangles, with a dotted line showing the means before-after cream application). The data show the mean values averaged over both cream tests and upper and lower 95% confidence intervals of the mean are shown at each mean point. Before cream application, water content was highest at the finger for both groups, but the young participants had significantly more water in the finger skin, as compared to the older participants at the finger. Cream application significantly increased skin water content over each skin site and for each group, apart from the young group on the finger and the older group on the arm.

Skin water content (skin electrical conductance) was analyzed by ANOVA using age group (younger n = 22, older n = 21), site (finger, arm, cheek), cream application (before/after), and cream type (A/B) as variables. Clear significant differences between water content were found between the skin sites (F(2, 491) = 266.9, p < 0.001, η^2^_p_ = 0.52), where finger > cheek > arm (all p > 0.001), as shown in Fig. 2. The older participants had drier skin than the younger participants, as found in a significant group × body site interaction (F(2, 491) = 37.5, p < 0.001, η^2^_p_ = 0.13), but Tukey-corrected post-hoc tests showed that this age group difference was only significant at the finger (p < 0.001, Cohen’s d = 1.8; arm p = 0.977, cheek p = 0.829). There was also a significant three-way interaction between age group, before/after cream application, and body site (F(2, 491) = 5.7, p = 0.004, η^2^_p_ = 0.03). Cream application significantly increased hydration (F(1, 491) = 87.7, p < 0.001, η^2^_p_ = 0.15) over each skin site and for each group (Tukey-corrected post-hoc tests all p < 0.013), apart from the younger group on the finger (p = 0.690) and the older group on the arm (p = 0.151) (Fig. 2). There was also a significant difference between the hydration given by each cream (F(1, 491) = 4.9, p = 0.033, η^2^_p_ = 0.01), where Cream A provided an overall 4.6% increase in hydration, as compared to Cream B, which increased water content by 4.2%.

### Tactile perception tests: skin monofilament detection sensitivity

**Figure 3:**
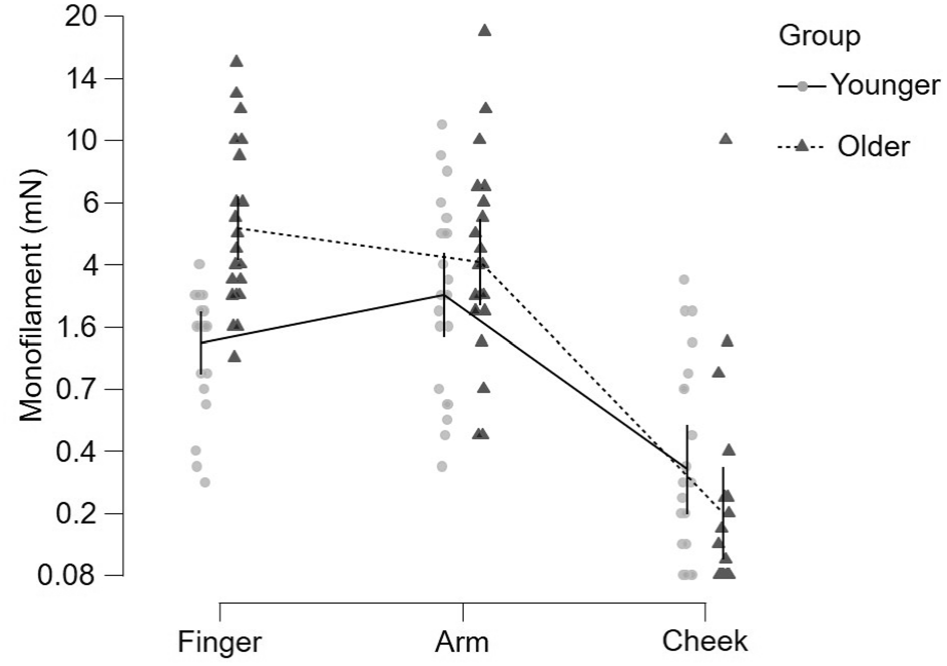
Tactile detection sensitivity over age groups at different skin sites. The monofilament tactile force detection (in mN) was measured in 43 participants (young group = 22, shown as filled lighter gray circles, with a continuous line showing the means between skin sites; older group = 21, shown as filled darker gray triangles, with a dotted line showing the means between skin sites) on glabrous finger skin and on hairy arm and cheek skin. The data show the mean values averaged over before and after cream application and for both creams and upper and lower 95% confidence intervals of the mean are shown at each mean point. There were significant main effects of age group and skin site, and an interaction between these, where there was only a significant difference between force detection threshold between the younger and older group at the finger.

Tactile detection thresholds were assessed by monofilament detection and repeated measures ANOVA were conducted with group (younger n = 22, older n = 21), site (finger, arm, cheek), cream application (before, after), and cream type (A/B) as variables. Monofilament thresholds were significantly different between body sites (F(2, 492) = 197.3, p < 0.001, η^2^_p_ = 0.45), where significantly lower (i.e. better) tactile detection was found on the cheek (Tukey corrected post-hoc tests: cheek vs. finger p < 0.001, cheek vs. arm p < 0.001, finger vs. arm p = 0.834), as shown in Fig. 3. Further, there was a significant interaction effect of age group by body site (F(2, 492) = 21.2, p < 0.001, η^2^_p_ = 0.08). This reflects that tactile detection was lower (better) only on the finger in the younger group (p < 0.001), but there was no difference between age groups for the hairy skin (arm p = 0.407, cheek p = 0.113) (Tukey corrected post-hoc tests). Conversely, there was no significant effect of cream application (F(1, 492) = 0.19, p = 0.660) or cream type (F(1, 492) = 0.12, p = 0.733) on tactile detection and none of the interactions were significant.

### Tactile perception tests: finger tactile spatial discrimination

**Figure 4:**
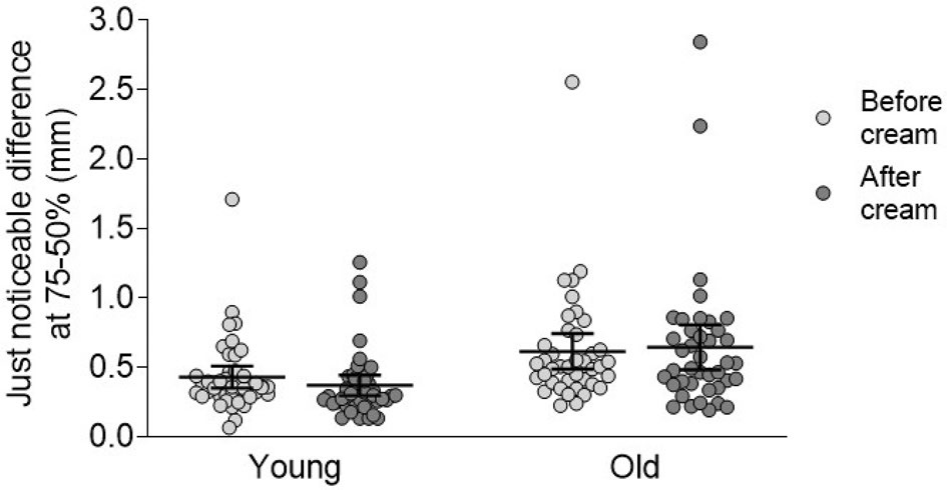
Finger tactile discrimination capacity in the younger and older groups before and after cream application. Participants discriminated between striated plates that had different spatial periods. A significant main effect of age group was found, where the younger group (n = 22, left side of graph) had better discrimination than the older group (n = 20, right side of graph), but no difference was found before (lighter gray circles) or after (darker gray circles) cream application. The black lines show the means for each age group, for before and after cream application and upper and lower 95% confidence intervals of the mean are shown.

We examined the ability to discriminate spatial gratings using active touch with the index finger. Plates with regularly spaced grooves of different spatial periods were presented in pairs, with a fixed reference plate, and plates of either a larger or smaller spatial period. We fitted psychophysical curves to the percentage of errors made in the tactile spatial discrimination task, to measure the capacity of participants to judge differences between the spatial gratings. From the psychophysical curves, we extracted the just noticeable difference (JND; 75–50%). One older participant was excluded from this analysis as the JND could not be determined, due to a lack of successful discrimination at the largest spatial differences. Using ANOVA, we found only a significant effect of age group (F(1, 160) = 16.6, p < 0.001, η^2^_p_ = 0.09), revealing better tactile discrimination capacities for the younger (n = 22) compared to older (n = 20) group, as seen in Fig. 4. No effect of cream application or type was found, nor any significant interactions. On average, for a just noticeable difference (75–50%), the younger group required at least 0.40 mm (± 0.03 SEM, variance 0.06) additional inter-band spacing to distinguish between the spatial gratings, whereas the older group needed at least 0.63 mm (± 0.05 SEM, variance 0.20).

### Tactile perception tests: pleasantness of arm stroking

**Figure 5:**
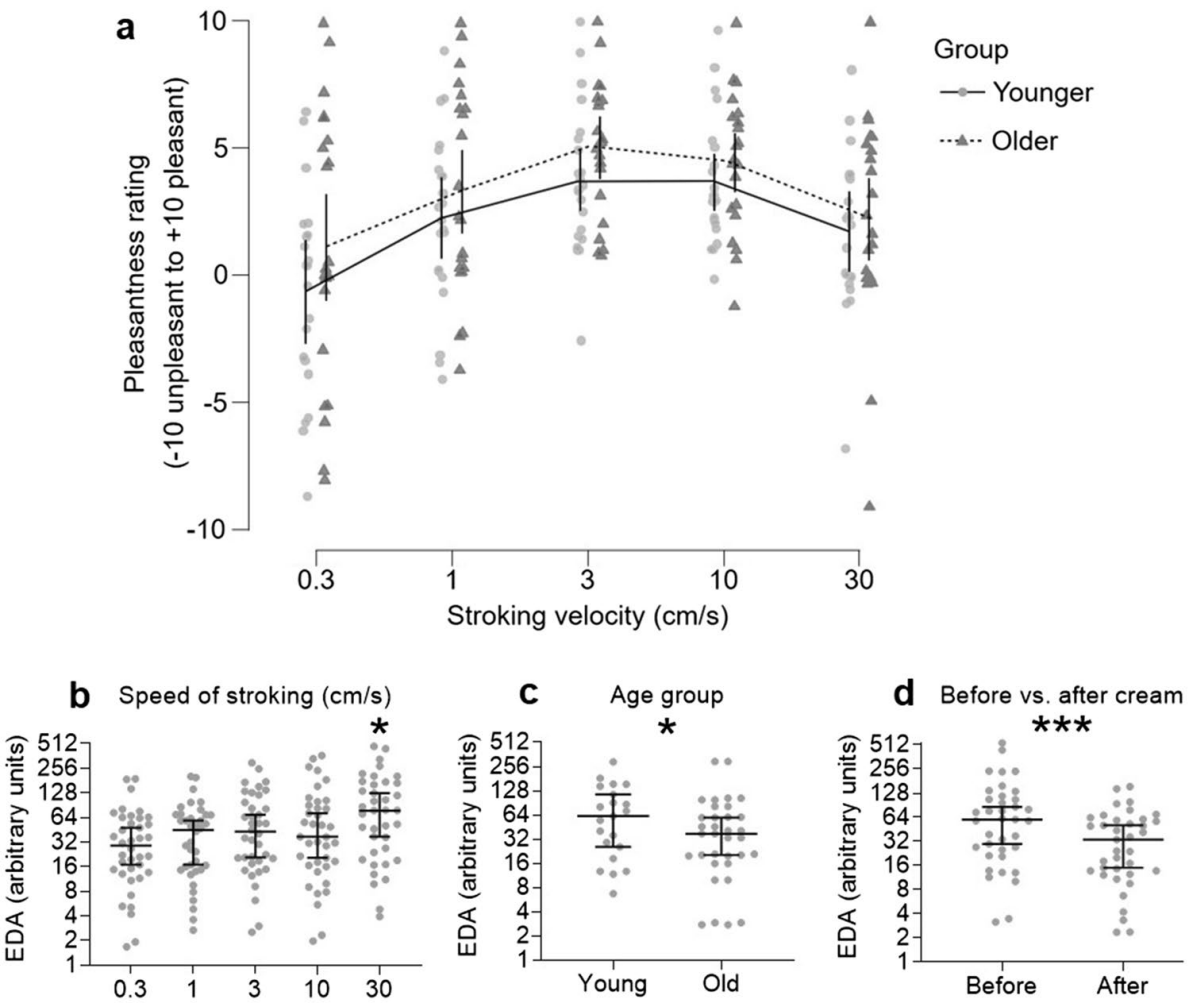
Pleasantness ratings over different velocities for the younger and older groups and electrodermal activity over the stroking conditions. Participants’ arms were stroked at different velocities before and after cream application. (**a**) Pleasantness ratings (range − 10 unpleasant to + 10 pleasant) for the younger (n = 22, filled gray circles, with the means connected as a continuous line) and older (n = 19, filled black triangles, with the means connected as a dotted line) groups over each stroking velocity (presented as a categorical log10 scale), showing a significant effect of age group and velocity. The lines showing the means also show the upper and lower 95% confidence intervals of the mean. During the stroking, electrodermal activity (EDA) was measured and significant differences were found for (**b**) stroking velocity, (**c**) age group, and (**d**) before and after cream application. As the data were highly skewed, medians are shown with 95% confidence intervals of the median, and each sub-figure y-axis is presented on a log2 scale, for better visualization of the data.

Tactile pleasantness was assessed through ratings of stroking the forearm at different speeds, for each age group (younger n = 22, older n = 21), before and after cream application, and with each cream type (A/B). ANOVA showed a clear main effect of stroking velocity on pleasantness ratings (F(4, 410) = 18.0, p < 0.001, η^2^_p_ = 0.15; Fig. 5a), where pleasantness was lowest during slow stroking (0.3 cm/s) and significantly less pleasant than all other velocities tested (post-hoc Tukey tests all p < 0.017). Pleasantness peaked around 3 cm/s stroking (no significant difference between 3 and 10 cm/s stroking, p = 0.99) and decreased again for faster stroking (30 cm/s, significantly different compared to all other stroking velocities (all p < 0.017), except for 1 vs. 30 cm/s p = 0.135). Additionally, there was a significant main effect of age group on tactile pleasantness (F(1, 410) = 8.9, p = 0.003, η^2^_p_ = 0.02), where the older group found the stroking more pleasant than the younger group (Fig. 5a). There was no effect of cream application or type, nor any interaction effects.

We also measured electrodermal activity (EDA) during the stroking paradigm, where we gained data from all 22 younger participants, but due to equipment failure, we only gained data from 19 older participants. A significant main effect was found for the speed of stroking (W = 28.7, p < 0.001), where EDA increased with stroking speed (Fig. 5b). Corrected post-hoc tests showed that stroking at 30 cm/s produced significantly more EDA than for the rest of the speeds (all p < 0.026). Age group also showed a main effect where the older participants has less overall EDA (Kruskal–Wallis W = 15.5, p < 0.001; Fig. 5c). Further, we found a significant effect of before/after cream application, where post-cream EDA was much lower (W = 39.5, p < 0.001; Fig. 5d).

### Tactile perception tests: ratings of cream characteristics

**Figure 6:**
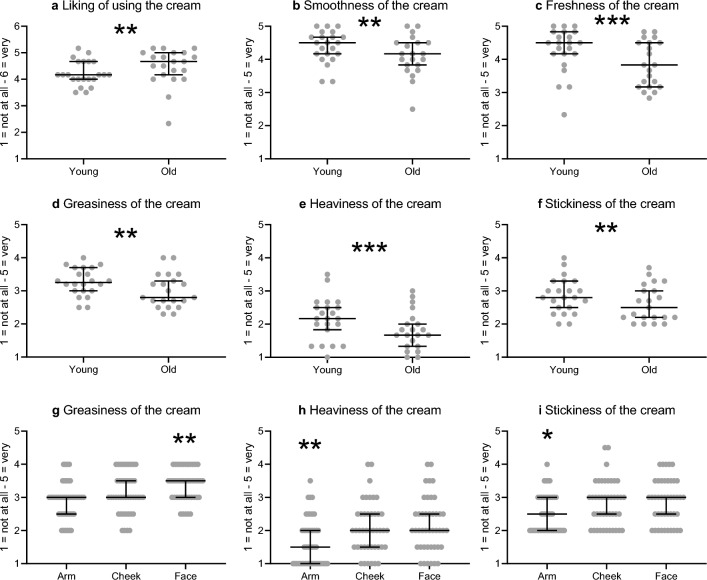
Perceptual ratings of cream characteristics during use. Median ratings (with 95% confidence intervals of the median) of individual participant datapoints are shown for each age group (younger n = 22, older n = 21 participants) for six perceptual measures of cream use. For (**a**) to (**f**), ratings are shown for differences between the age groups on: (**a**) how much the participants liked using the cream, (**b**) the smoothness of the cream, (**c**) the freshness of the cream, (**d**) the greasiness of the cream, (**e**) the heaviness of the cream, (**f**) the stickiness of the cream. For (**g**) to (**i**), ratings are shown for differences between ratings over skin sites on: (**g**) the greasiness of the cream, (**h**) the heaviness of the cream, (**i**) the stickiness of the cream. Asterisks for significant differences on Kruskal–Wallis tests are denoted as **p < 0.01, **p < 0.001, where for (**g**) face ratings were significantly lower than for the arm, (**h**) arm ratings were significantly lower than on the cheek and face, (**i**) arm ratings were significantly higher than on the cheek and face. Note that confidence intervals that do not appear above and/or below mean that the interval is at the median.

We present below the findings from six questions that were related to the perception of the cream during use, over the three sites (arm, cheek, whole face), for the younger (n = 22) and older (n = 21) groups.

i. *Did you like using the cream?* The participants liked using the creams and the ratings revealed significant differences between age groups (Kruskal–Wallis W = 9.3, p = 0.002; Fig. 6a), where older participants liked the creams more.
ii. *How smooth was the cream?* The participants rated the texture of the creams as very smooth, but the younger group rated the creams as significantly smoother than the older participants (Kruskal–Wallis W = 6.7, p = 0.010; Fig. 6b).
iii. *How fresh was the cream?* The participants rated the creams as fresh on application and the younger participants rated the creams as significantly fresher than the older group (Kruskal–Wallis W = 15.8, p < 0.001; Fig. 6c).
iv. *How greasy was the cream?* The participants rated the creams as moderately greasy. A significant effect of age group was found (Kruskal–Wallis W = 10.3, p = 0.001; Fig. 6d), where the younger group rated the creams as being greasier than the older participants (Fig. 6b, left). A significant effect of body site was also found (Kruskal–Wallis W = 14.8, p < 0.001; Fig. 6g), where the whole face application was found to be felt as significantly greasier than on the arm (corrected Dunn’s post-hoc test p = 0.006), as shown in Fig. 6b, right.
v. *How heavy was the cream?* The participants felt that the creams were light on the skin, but there was a significant difference between the age groups, where the older group felt it to be significantly lighter on the skin than the younger participants (Kruskal–Wallis W = 7.1, p = 0.008; Fig. 6e). There was also a significant difference between the feeling of how heavy the creams were between body sites (Kruskal–Wallis W = 14.2, p < 0.001; Fig. 6h). Here, the creams were felt as significantly lighter on the arm than on the cheek (p = 0.003) or on the face as a whole (p = 0.009) (corrected Dunn’s post-hoc tests), as shown in Fig. 6c, right.
vi. *How sticky was the cream?* The participants rated the creams as moderately sticky, but there was a significant difference of age group (Kruskal–Wallis W = 8.2, p = 0.004; Fig. 6f), where the younger group found the creams to be stickier than the older group (Fig. 6d, left). There was also a significant difference between body sites (Kruskal–Wallis W = 9.6, p = 0.008; Fig. 6i), where the cream on the arm felt significantly less sticky than when it was applied to the cheek (p = 0.029) or whole face (p = 0.002) (corrected Dunn’s post-hoc tests), as shown in Fig. 6d, right.

### Selective correlations between measures

**Figure 7:**
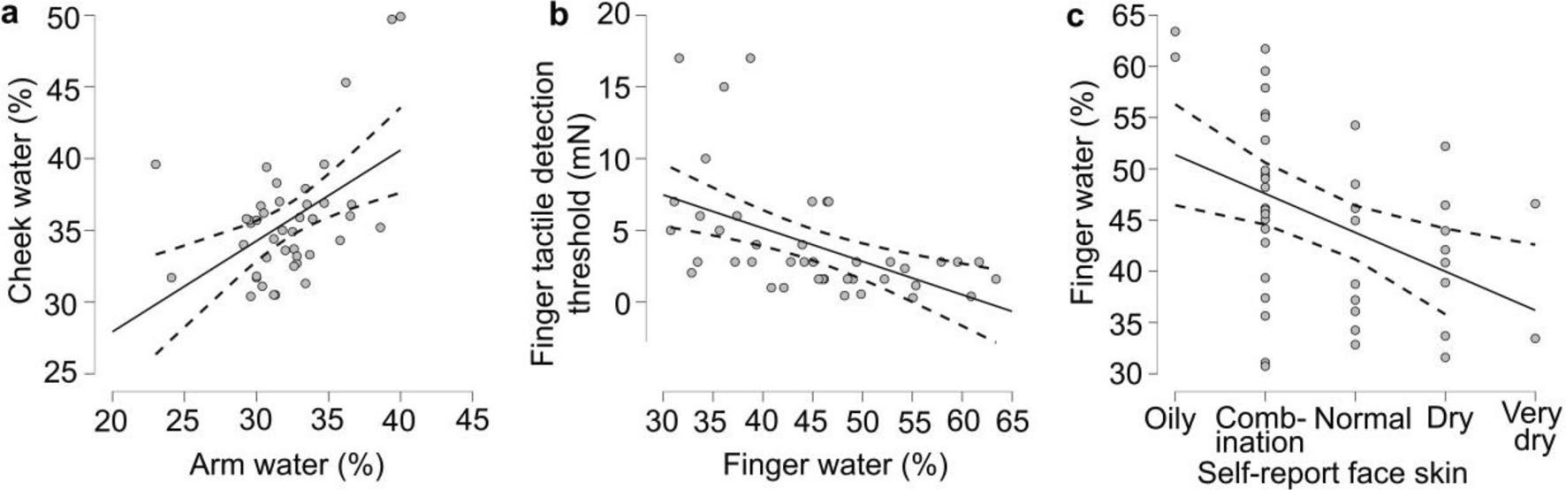
Significant correlations between selected measures of skin water content, tactile detection, and self-reported face skin classification. (**a**) Correlation of skin water content at the forearm and cheek (Pearson p < 0.001). (**b**) Relationship between finger water content and tactile detection threshold (Pearson p < 0.001). (**c**) Correlation between self-reported face skin dryness classification with finger water content (Spearman p = 0.005. Each dot represents a participant (n = 43), with the solid line showing the correlation and the dotted lines the upper and lower 95% confidence intervals.

On the basis of the above findings, we conducted exploratory analyses to test whether there were relationships between specific measures. In Fig. 7a, we investigated if the water content of the finger, arm, and cheek were correlated, as there are differences between the regulation of water between hairy and glabrous skin. We found that there was only a significant relationship between the water content of the hairy skin, i.e. arm and cheek (Pearson’s R = 0.49 p < 0.001, n = 43, Fig. 7a). There was no significant correlation between finger and arm water content (R = 0.20 p = 0.194, n = 43), nor between finger and cheek water content (R = 0.18 p = 0.256, n = 43).

We also investigated whether the water content of the finger was related to touch measures, as the finger capacity was especially affected by age. We found that finger water content was significantly correlated with tactile detection (Pearson’s R = -0.50, p < 0.001, n = 43, Fig. 7b), but not with tactile spatial discrimination (Pearson’s R = -0.14, p = 0.390, n = 42). Therefore, the tactile detection appeared to be related to finger hydration.

As there was no difference in the water content between the younger and older group on the cheek, but the older participants self-reported having drier face skin, we explored whether there was an association between the objective measures of water content and the self-perceived dryness of the face. No significant relationship was found between face skin dryness classification and cheek water content (Spearman’s R = 0.12, p = 0.440, n = 43). However, we found a significant correlation between the finger water content and the self-reported face skin classification (Spearman’s R = − 0.42, p = 0.005, n = 43) (Fig. 7c). Thus, the facial skin perception was driven more by the characteristics of the finger than the face itself.

## Discussion

We conducted a variety of tests to investigate differences in touch between glabrous finger skin, and hairy forearm and cheek skin, finding differences between sites, with age, and cosmetic cream application. Overall, our results from self-perceptual measures, psychophysical tests, physiological responses, and perceptual ratings, show that there are significant changes with age on the capacity of the skin of the finger (reduced natural hydration levels, detection, and discrimination capacities), but the hairy skin (arm and cheek) are less affected by age. Here, we raise the possibility that the reduction in finger capacity with age affects the self-perception of the skin (face and arm) as well as the evaluation of the cream characteristics (lighter and smoother). Using a cosmetic product just before performing the tactile tasks increased hydration levels, but this was insufficient to improve the present task performance. Hence, complex mechanisms seem to interact in the self-evaluation of our own skin, where finger capacity seems to play a determinant role.

### Glabrous finger skin tactile capacity decreases with age

We found large differences in our measures at the finger, where the skin of all participants had an overall higher water content than the hairy skin (arm and cheek), but the older group had significantly less hydrated finger skin than the younger group. In addition, finger tactile detection and spatial discrimination were also reduced with age, reflecting general capacity decreases of the finger with age. Water content of the skin is determined by several sources, including the subcutaneous fluid content, sweat gland activity, and environmental humidity^29^. The device we used measured skin water content via electrical skin conductance^30,31^, which quantifies the water content at the skin surface, and is strongly influenced by the output of eccrine sweat glands^32,33^. A study by Wildgoose et al.^30^ found skin conductance significantly decreased with age, as per our results. However, they found no significant main effect of skin conductance between six body sites, with no specific difference on the finger, but this was only in 16 participants (6 older). In our larger sample, the effect of aging on skin water content was strongest and significant only on the finger, thus showing a somewhat specific effect. With aging, there are structural changes in eccrine sweat glands, as well as a decrease in sweat output^34,35^. This may account for the decrease in skin water content, especially as these sweat glands are densest in the fingers and toes, although gland density can vary greatly between individuals^36^. Therefore, the decline in skin capacity with age, may be directly related to decreases in sweat output and restoring the water content may provide advantages in touch.

As well as changes in skin water content, we found a significant decline in tactile detection with age. However, this was only on the finger. Further, there was a significant correlation between finger detection sensitivity and water content, where the higher the finger hydration, the lower the touch detection threshold. This suggests that the decline in neural encoding capacity co-occurs with skin structural changes, where both likely impact each other^37,38^. Spatial discrimination with the index finger was also reduced, which could be due to many factors, including task difficulty and decline of cognitive systems with age^38^. Additionally, the older group showed increased variability^2^, which could be due to many factors, including task difficulty and decline of cognitive systems with age. The increased variability of the older group may have caused the lack of significant correlation with the finger water content and spatial discrimination threshold, which could be specifically explored in future work.

In all, this shows that the finger skin is subject to large, detrimental changes with age that can directly reduce our capacity to interact with our environment. Previous work has highlighted that the fingers and toes are especially vulnerable to tactile decline with age^21^, which again may link to sweat gland density, as well as to continued usage and exposure. Therefore, increasing skin water content of the hands, as well as the feet, could be advantageous for object manipulation and locomotion, respectively. Although we did not find a significant effect of cream application on tactile detection or discrimination, it may be that this application is insufficient to modify touch in the very short-term. On the other hand, previous work has shown that tactile discrimination can improve very soon after application^2,28^ and also over days^28^ and after a month^27^. Thus, it may be that the cream needs to sit on the skin for longer and that continued hydration is required. Based on this, it is pertinent to explore whether well-hydrated foot skin could be a way of increasing tactile signals from the feet and reducing falls in the elderly^39^.

### Hairy skin properties are preserved with age

Although the finger was highly affected by age, no effect was found for skin water content or tactile detection for the hairy cheek and arm skin. As per previous work^11,13^, we found that the cheek skin was highly sensitive to touch, with numerous participants reaching the lowest monofilament level. This suggests preserved capacity of facial skin with age. However, in the self-reports of face skin perception, older participants found their skin to be drier. Further, there was no correlation with these ratings and the cheek water content. Although the face skin may change anatomically, with the appearance of wrinkles and decrease in elasticity, sensory function is preserved, providing us with excellent tactile feedback throughout life^21^. Thus, a cosmetic cream may hydrate and preserve the face skin, but a deep hydration may not necessarily be required at this location, and such creams could focus on improving the anatomical changes in face skin with age. However, as face cream use increased in the older group, this may have also contributed to maintaining their facial skin hydration and tactile sensitivity.

Cosmetic creams are applied to the face using glabrous hand skin, thus the surface of the finger skin may influence how we perceive our facial skin. In the older group, finger skin had significantly lower water content, thus was likely to have a drier and rougher texture. The application of a cream by a rougher surface may lead to the perception that the skin receiving the cream also feels rough^26^. We explored the link between finger skin hydration and self-perception of facial skin and found that lower finger water was associated with perceived facial dryness. Thus, we hypothesize that the finger is used as a probe to assess skin properties and can influence the perception of self-touch.

### Touch appreciation changes with age

Using a classic forearm skin stroking paradigm, we independently replicated the finding that the pleasantness of all stroking velocities significantly increases with age^24^ in a different European country. Only a handful of studies have investigated pleasant touch perception in older age (> 60 years^40^) and various reasons for this increase have been proposed, from potential changes all along the path, from peripheral encoding to central interpretation. In the previous work, Sehlstedt et al.^24^ found that pleasant touch appreciation increased with age, but not olfactory pleasantness, suggesting that the mechanism is not from a general increase in sensory appreciation. Presently, we also recorded EDA during tactile stroking, which measures arousal, and found higher EDA with faster stroking. Contrary to pleasantness, ratings of tactile intensity typically increase with stroking speed^24,41^, hence this may lead to higher arousal. Further, EDA during arm stroking decreased with age, which may be linked to decreased skin sympathetic nervous system output^42^ to sweat glands. A previous study found decreased EDA and increased affective appraisal of negative pictures with age, showing the same trend that arousal decreases with age, but affect increases^43^. Another finding was that EDA responses to stroking were lower after cream application. This effect may have been linked to the repeated gentle touch to the arm (brushing and cream massage), which is inherently pleasant and calming, and/or changes in skin friction^28^.

We also asked the participants questions about how they evaluated different characteristics of using the creams. We found differences between skin site and with age. Linked to pleasantness, the older participants liked using the creams more than the younger group. Although this may have been for a variety of reasons (e.g. cosmetic needs, motivation), they may have an overall higher appreciation of the affective aspects of touch with age. The participants also rated characteristics of the creams, where the older group found cream application to be less greasy, lighter, and less sticky than the younger participants. This could reflect that the evaluation of the creams is biased by finger dryness. Taken together, the general appreciation of the cream (liking) increased, while the evaluation of the sensory aspects (greasiness, heaviness, stickiness) decreased, which could reflect a general change in the balance between affective and sensory tactile evaluation with age. Further, for all participants, the sensory ratings were greater on the face than on the arm. This may have been due to a number of differences between the face and arm, such as the density of mechanoreceptors, tactile sensitivity, cortical representations, and the important link between hand-face contact^8,44^.

### Limitations and future perspectives

We investigated two age groups to compare differences in touch with age; however, our populations were restricted to healthy females. This may limit the generalizability of our work and a wider population may show more variability, such as through different life experiences. We used a number of different tests, which allowed us to obtain much data, but the tests were adapted to yield data quicker. For example, we used a short tactile detection task using monofilaments, but this can be conducted in a number of ways, which may give more accurate thresholds^45^. However, we had very low detection thresholds, therefore even more sensitive touch tests may be required, especially on the face, which was highly sensitive. Although lighter force monofilaments are difficult to make and use, there are plenty of other tactile tests that could be carried out across the skin to assess different tactile perceptions, such as vibration perception and spatial discrimination of two points^45^.

There are certain evident changes in the touch system with age, such as a general decline of tactile capacity of the fingers. Touch is highly heterogenous across the body, as evidenced by differences in mechanoreceptor type and density^8^, although hairy skin capacity was far less affected with age. However, we only tested hairy arm and face skin, therefore we cannot generalize to the rest of the body. Further, sweat control and optimal skin hydration are key factors in object manipulation^46,47^, thus with decreased sweating with age and drier glabrous skin, older people are at risk of impaired hand sensorimotor control, which may also be applicable to the feet and locomotion, to prevent falls. Multiple factors could account for these differences with age, including peripheral and central neural changes, hormones, biophysical changes of the skin, diseases, life experiences, and environmental exposure. Although skin changes with age, its protection is important and linked to tactile performance. As well as other possible strategies (e.g. hormonal treatment), adequate skin care is regarded as a major strategy for maintaining skin barrier integrity and health. Hydrating the skin with a cosmetic product is a common and pleasant daily habit, and as such, appears as a relevant skin rehabilitation strategy compensating for the neural loss in aged individuals, with important outcomes on the well-being. In addition, this could be applied in improving self-perception of body image with aging, to ultimately increase self-esteem, and to create new opportunities for cosmetic products^48^. Altogether, this paves the way for skincare products formulated with ingredients intended to optimally maintain sensory function.

## Conclusion

We investigated how tactile perception in glabrous and hairy skin changes with age and the application of a cream in females. We highlight differences between discriminative and affective touch with age, where discriminative tactile capacity decreases, but only on the glabrous finger, and the positive, pleasant aspects of touch become stronger. We emphasize the importance of the hand as a probe for exploration of our environment, others, and our own body. Indeed, our hands are fundamental in self-touch, where their capacity impacts the perception of the body, thus their preservation is important in self-care. For example, changing simple habits, such as moisturization after hand washing and regular hydration of the hands and feet, could improve quality of life and well-being.

## Methods

### Participants

A total of 44 female participants were recruited for the study from two age ranges. For the younger group, 22 participants (median age: 24 years, range: 20–28 years, 3 self-reported left-handed) completed both experiments. For the older group, 22 participants were then recruited in the screening session, but one was found to have significant sensory deficits, warranting exclusion from the main study, thus 21 participants were included in the older group (median age: 70 years, range: 65–75 years, 1 left-handed). Exclusion criteria were: current pregnancy or lactation, delivery or breastfeeding within the previous six months, a history of neurological, psychiatric, or dermatological disorders, or clinically significant peripheral neuropathy. The study was approved by an ethical committee (Comité de Protection des Personnes Est-III) and they were paid for their participation. The study was performed in accordance with national guideline/regulations and the Declaration of Helsinki, apart from pre-registration in a database, and written informed consent was gained.

### General procedure

Participants were first invited to a screening session. The experimenter gave them further details about the study, answered any of their questions, went through the consent procedure and familiarized them with the experimental set-up used for the study. Two experimental sessions lasting 2.5 h each were then planned with at least a week of delay between each. In each session, one of the two cosmetic creams was tested (Cream A or Cream B). Both test formulations were cosmetic products available on the market, with safety certificates. Cream A was an inverse emulsion (oily continuous phase) and cream B was a direct emulsion (continuous aqueous phase). The products were in identical pots and were lightly perfumed in the same way. The order of the creams was counter-balanced and pseudo-randomized between each age group and the tests were double-blinded (i.e. neither the experimenter nor participant knew which cream was being applied). The participants were asked not to use cosmetic products on the skin of the face, arms, and hands on the day of the experimental visit.

Each session was composed of pre-tests, followed by cream application, and then post-tests. The same tests were performed in the same order for the pre- and post-tests. A series of measurements (skin water content, tactile detection) and tests (evaluating discriminative and affective touch) were conducted. Questionnaires (e.g. evaluating the liking of the creams) were also employed after cream application.

The participants were tested in a quiet room, with a constant temperature of 22 °C. For all tests, they wore an eye mask and noise cancelling headphones (Bose, Framingham, MA). The measures of skin water content and tactile detection sensitivity were conducted at three skin locations: on the glabrous skin of the right index finger (middle of the distal phalanx), on the left forearm (hairy skin, dorsal aspect, at 10 cm from the wrist fold), on the left cheek (hairy skin, middle of the fleshy part). The test of tactile discrimination was performed with the tip of the right index finger. The test of tactile pleasantness was performed at the left forearm, which was supported by a vacuum cushion to allow a standardized and relaxed position during stroking. Surface electrodes were strapped around the fingers of the left hand to record electrodermal activity (EDA) during the tactile pleasantness task.

The participant sat behind a table, with the experimenter on their right side. After the procedures had been briefly re-explained, dots were marked at the three skin locations of interest. The eye mask and the headphones were then placed on the participant. The test of tactile detection was immediately followed by the water skin content measurements at each test location. Skin location order was randomized for each participant, and, between the pre- post-test sessions. The test of discrimination was then performed. The cream was then applied to the skin. To achieve so, the participant first watched a video describing how to rub the skin in a standardized manner. The experimenter then applied 0.05 ml of cream using a micropipette to give four spots, either on the left forearm (within an area of 10 cm around the initial marked dot) or on the left index finger (distal phalanx). The participant then rubbed the cream using their right index finger on the left forearm (cream on the arm); or on the left cheek (cream on the left finger). The order of body site was randomized. A 5 to 10 min pause was then taken within which the participant answered questionnaires about the usage of the creams. The participant then sat back at the initial table and the post-tests began. At the end of the session, the participants freely applied the cream to their whole face and completed the same questionnaires another time (not shown). Short pauses were regularly proposed after each test.

### Measures

#### Self-reported measures

*Skin type and moisturizer use*. In the screening session, the self-reported face skin type (oily/combination (oily nose and forehead skin)/normal/dry/very dry) and body skin type (normal/dry/very dry) was noted for each participant. The following self-reported moisturizer uses were also gathered: frequency of day cream use, frequency of night cream use, frequency of body cream use (all on the scale: everyday/4–5 times per week/2–3 times per week/once a week/once a fortnight/less often/never).

#### Physical skin characteristics: water content

A handheld device (Skin Analyser, Hurrise) was used to measure the water content of the skin before and after cream application. It is pressed on the selected skin site (index finger, arm, cheek) for a few seconds and analyses bioelectric impedance to provide measurements of the skin conductance (reciprocal of resistance), expressed as a percentage^31^.

#### Tactile perception tests: skin monofilament detection sensitivity

A calibrated monofilament detection task was used to assess skin tactile sensitivity before and after cream application. A total of 13 calibrated monofilaments over a wide range from 78 mN (8 g) to 0.08 mN (0.008 g) were applied (full range: 78, 59, 39, 20, 14, 10, 6, 4, 1.6, 0.7, 0.4, 0.2, 0.08 mN) to the selected body site (index finger, arm, cheek). The test began using the 39 mN monofilament and the experimenter applied this to the skin three times. We used a descending force detection paradigm^45^, where the participant had to say ‘top’ when they felt touch with the stimulus. If there were three correct responses, the monofilament level decreased by two (e.g. next would be 14 mN). Next, the adjacent higher level was tested (i.e. 20 mN). If an error occurred, the adjacent higher force level was applied. The test was terminated when two errors were made at one level and the monofilament at the level before this was defined as the threshold (e.g. if two errors were made at 4 mN, the threshold level was 6 mN). If the participant reached the lowest (hardest) level (i.e. 0.08 mN) with no errors, this was defined as the threshold.

#### Tactile perception tests: finger tactile spatial discrimination

Participants were asked to explore pairs of plastic plates, composed of regularly spaced grooves with distinct spatial periods from 3.6 to 6 mm, by moving their right-hand index fingertip once across the plate. The task was a two-alternative forced choice task of discrimination^27^. The sheet with a median spacing of 4.8 mm was used as the reference. With each paired presentation, the reference was compared to one of the 10 other sheets. The participant had to say whether the second sheet had a larger inter-band spacing than the first (yes/no). Between sheets, the inter-band space varied in 0.2 mm steps on each side of the reference, and by 0.4 mm for the two extreme sheets. The participants were instructed to explore each plate once, using the right index finger, from top to bottom (thus with the grooves being oriented perpendicularly to the finger displacement), at their own preferred and constant speed. The two extreme plates were presented two times in total, and the other plates were presented four times, which is justified by the ease in differentiating the former plates from the reference. Thus, in total, the participants had to compare 36 reference/test pairs. The order of presentation was randomized within and between pairs. This psychophysical examination took about 15 min.

We measured the spatial acuity in mm, hence, representing the amount of increase in inter-band space needed to reliably detect a difference (75% correct responses) compared to the reference (just noticeable difference, JND, 75–50%). This was determined using classic psychometric procedures (percentage of correct responses fitted by a cumulative Gaussian function) and the PALAMEDES toolbox^49^ in MATLAB (The Mathworks, MA). For the analysis and construction of the psychophysical curves, responses were sorted by if the participant said that the test plate was more spaced than reference plate.

#### Tactile perception tests: pleasantness of arm stroking

A rotary tactile stimulator (RTS; Dancer Design, St Helens, UK) was used to deliver controlled brush strokes at a predetermined force, direction, and speed to the skin sites in question, using custom written scripts in LabVIEW (version 2010; National Instruments, Austin, TX). A soft brush moving across the skin was used as a pleasant stimulus (5 cm wide goat hair artists’ brush). A total of five velocities (0.3, 1, 3, 10, and 30 cm/s) were tested three times per skin site, in a pseudo-randomized order. The stimulator was placed over the middle of the forearm. The force applied by the brush was calibrated at 0.4 N and the stroking was delivered in a proximal to distal direction. This is the typical approach used for tactile pleasantness assessment (e.g. see^13,50^). After each brush stroke, the participant rated the pleasantness of the sensation using a visual analogue scale with the end anchors “Unpleasant” to the left and “Pleasant” on the right. There was a 10 s pause between strokes. Analyses were conducted on the averages of the three stroking repeats, giving three mean stroking velocity pleasantness data points per skin site, per participant, before and after cream application. During the tactile pleasantness task, measures of emotional arousal were obtained using a PowerLab system (ADInstuments, Dunedin, New Zealand), recording EDA via a Galvanic Skin Response Amp with bipolar finger electrodes using LabChart software (version 7; Dunedin, New Zealand). EDA data were treated off-line using MATLAB (version 2021b; The Mathworks, MA). Baseline-corrected measurements were taken from an 8 s time period after the start of the stroke for each trial, where the area under the curve was obtained and averaged per stroking velocity, per participant.

#### Perception of the creams

As the study was aimed at providing insights into the perception of the creams, a total of 19 questions were posed to the participants after each cream application. As many of these questions relate specifically to the industrial partner (e.g. ease of application, liking of the smell, novelty of the texture), we only present the data from questions that are on the sensory perception of the creams. These six unipolar→r questions were: (i) Did you like using the cream? (1 = not at all to 6 = very much), (ii) How smooth was the cream? (1 = not at all smooth to 5 = very smooth), (iii) How fresh was the cream? (1 = not at all fresh to 5 = very fresh), (iv) How greasy was the cream? (1 = not at all greasy to 5 = very greasy), (v) How heavy was the cream? (1 = not at all heavy to 5 = very heavy), (vi) How sticky was the cream? (1 = not at all sticky to 5 = very sticky).

### Statistics

Statistical analyses were conducted using JASP (version 0.17.2;^51^) and plotted with JASP and Prism (version 7; GraphPad, Boston, MA). We used a number of methods to assess how to analyze the data. For each measure, we visually examined the data plotted as Q-Q plots, calculated descriptive statistics (e.g. minimum, maximum, standard deviation) and considered whether the data were fundamentally ordinal (discrete) or interval (continuous). We also used Shapiro–Wilk tests to test the normality of the residuals.

Based on these assessments, we considered the self-reported measures of skin measures and moisturizer use and the perception of the creams to be ordinal data, thus used non-parametric statistics. For the self-reported skin measures and moisturizer use), Mann–Whitney (U) independent sample tests were used to compare differences between the older and younger groups. For the perceptual ratings of the creams, we used Kruskal–Wallis (W) tests, with multiple comparison corrected Dunn’s tests when comparing more than two variables. The EDA measures were skewed and the residuals of these data were not normally distributed (Shapiro–Wilk all p < 0.001), thus non-parametric Kruskal–Wallis (W) tests were also used to analyze these data.

Also based on the above assessments, we used parametric analyses for the skin water content, tactile detection sensitivity, tactile spatial discrimination, and for tactile pleasantness ratings. Repeated measures ANOVA was used to compare each measure, where post-hoc tests were carried out on significant main effects. For multiple comparisons, p-values were adjusted using Tukey corrections.

As we were interested in associations between certain measures, we performed selected correlations, which were corrected for multiple comparisons, between specific variables. When the variables obtained were interval data, we used Pearson’s correlation, and when the variables were ordinal, we used Spearman’s correlations.

Individual participant datapoints are presented in the figures, where feasible, with means (for interval data) and medians (for ordinal data) and the corresponding upper and lower 95% confidence intervals of the mean/median (CI). Estimates of effect size (partial eta squared, η^2^_p_ for ANOVA and Cohen’s *d* for between-means tests) are given, where applicable.

## Data Availability

The raw data are available in a permanent project repository on Open Science Framework at https://osf.io/znm2r/, where each measure is saved as an individual file.
