# Author Correction: Effects of skin moisturization on various aspects of touch showing differences with age and skin site

**DOI:** 10.1038/s41598-024-59646-8

**Published:** 2024-04-23

**Authors:** Mariama Dione, Roger Holmes Watkins, Jean‑Marc Aimonetti, Roland Jourdain, Rochelle Ackerley

**Affiliations:** 1 Aix Marseille Univ, LNC (Laboratoire de Neurosciences Cognitives – UMR 7291), CNRS, Marseille, France; 2 L’Oréal Research & Innovation, Aulnay‑sous‑Bois, France

Correction to: *Scientific Reports* 10.1038/s41598-023-44895-w, published online 20 October 2023

The original version of this Article contained an error in Figure 5, panel d, where the values were swapped round. The original Figure 5 and accompanying legend appear below.

**Figure 5:**
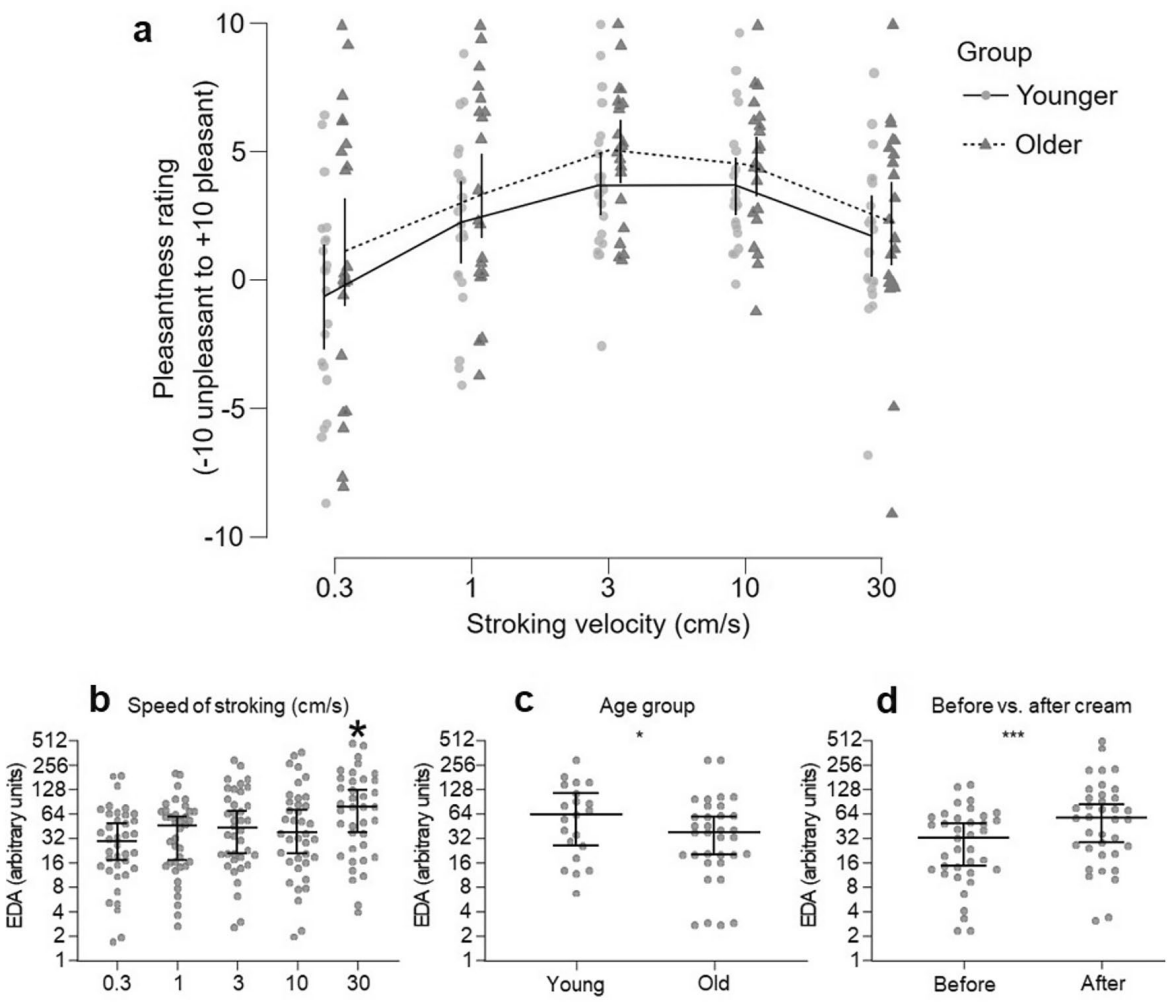
Pleasantness ratings over different velocities for the younger and older groups and electrodermal activity over the stroking conditions. Participants’ arms were stroked at different velocities before and after cream application. (**a**) Pleasantness ratings (range − 10 unpleasant to + 10 pleasant) for the younger (n = 22, filled gray circles, with the means connected as a continuous line) and older (n = 19, filled black triangles, with the means connected as a dotted line) groups over each stroking velocity (presented as a categorical log10 scale), showing a significant effect of age group and velocity. The lines showing the means also show the upper and lower 95% confidence intervals of the mean. During the stroking, electrodermal activity (EDA) was measured and significant differences were found for (**b**) stroking velocity, (**c**) age group, and (**d**) before and after cream application. As the data were highly skewed, medians are shown with 95% confidence intervals of the median, and each sub-figure y-axis is presented on a log2 scale, for better visualization of the data.

The original Article has been corrected.

